# A Systematic Review of Metabolomic and Lipidomic Candidates for Biomarkers in Radiation Injury

**DOI:** 10.3390/metabo10060259

**Published:** 2020-06-20

**Authors:** Elisabeth Vicente, Zeljko Vujaskovic, Isabel L. Jackson

**Affiliations:** Division of Translational Radiation Sciences, Department of Radiation Oncology, University of Maryland School of Medicine, Baltimore, MD 21201, USA; elvicente@som.umaryland.edu (E.V.); zvujaskovic@som.umaryland.edu (Z.V.)

**Keywords:** biomarker, radiation, metabolomic, lipidomic, citric acid, citrulline, creatine, uric acid, ceramide, diagnostic

## Abstract

A large-scale nuclear event has the ability to inflict mass casualties requiring point-of-care and laboratory-based diagnostic and prognostic biomarkers to inform victim triage and appropriate medical intervention. Extensive progress has been made to develop post-exposure point-of-care biodosimetry assays and to identify biomarkers that may be used in early phase testing to predict the course of the disease. Screening for biomarkers has recently extended to identify specific metabolomic and lipidomic responses to radiation using animal models. The objective of this review was to determine which metabolites or lipids most frequently experienced perturbations post-ionizing irradiation (IR) in preclinical studies using animal models of acute radiation sickness (ARS) and delayed effects of acute radiation exposure (DEARE). Upon review of approximately 65 manuscripts published in the peer-reviewed literature, the most frequently referenced metabolites showing clear changes in IR induced injury were found to be citrulline, citric acid, creatine, taurine, carnitine, xanthine, creatinine, hypoxanthine, uric acid, and threonine. Each metabolite was evaluated by specific study parameters to determine whether trends were in agreement across several studies. A select few show agreement across variable animal models, IR doses and timepoints, indicating that they may be ubiquitous and appropriate for use in diagnostic or prognostic biomarker panels.

## 1. Introduction

A nuclear accident or attack has the potential to produce large-scale mass casualties by exposing individuals to potentially life-threatening doses of ionizing irradiation (IR). Acute radiation sickness (ARS) occurs in humans at doses of total body irradiation (TBI) in excess of 0.5 Gy, with life- threatening bone marrow suppression occurring after exposure to 2 Gy or higher. The severity of ARS increases with radiation dose and quality (e.g., low or high linear energy transfer radiation) and is characterized by bone marrow suppression and/or failure, infection, hemorrhage, and severe acute anemia. Delayed effects of acute radiation exposure (DEARE), such as radiation pneumonitis or fibrosis, may manifest several weeks to months after exposure among individuals surviving through the ARS [1,2].

Following a nuclear event, both evacuation and evaluation of the affected population surrounding the epicenter will require medical triage. First assessment will include the location of the incident, radiation exposure associated symptoms, and physical examination to identify the at-risk population. Second, a point-of-care assessment of 2 Gy or greater will be subjected to further testing to identify those exposed to potentially-life threatening doses of radiation [3]. The timeline of testing would be from 1 to 7 days post-exposure based on the current concept of operations and to allow sufficient time to medically intervene in the course of the disease. Easily accessible bio-fluids, such as blood or urine, are essential in a mass casualty event because their collection is non-invasive, and they are easily acquired from critically injured and non-responsive individuals [4].

While one biomarker is unlikely to reflect the physiological response to radiation induced injury, the ideal panel of biomarkers would be diagnostic, prognostic, predictive, or pharmacodynamic. Briefly, a diagnostic biomarker is a simple test that will identify whether a person has been exposed to radiation. In contrast, a prognostic biomarker will determine the level of risk for injury development and progression independent of therapy. Prognostic biomarkers may aid in determining survivability, the severity of the injury, or even the levels of organ specific injury at the time of sampling. A predictive biomarker will have the same characteristic as prognostic and will further discriminate whether or how a person will respond to targeted therapy. Last, a pharmacodynamic biomarker will show a direct pharmacological effect of a therapy with evidence [5,6].

Both diagnostic and prognostic biomarkers used during early exposure testing will guide patient stratification gradings, such as (1) not exposed, (2) low or sublethal, (3) high or lethal exposure [7,8]. Evaluation of each individual is essential to provide the appropriate therapy; however, the panel of biomarkers and the precise method in which this will be executed has yet to be elucidated. For this reason, extensive effort has been made to identify biomarkers that may be used for initial testing [9]. A biodosimetry test will accurately estimate the absorbed radiation dose by quantifying key biomarkers per individual. More specific biomarkers may indicate whether an individual has received organ specific damage. The results, along with the duration of time from exposure will enable a more unique and individualized therapy.

The development of accurate and precise biomarkers of radiation injury is complicated by the understanding that an improvised nuclear device will most likely be composed of low- and high-dose-rate photon and neutron elements. Further, ingested radionuclides and delayed exposures due to ground shine or fallout may also result, exposing individuals to variable dose rates and qualities of radiation. High-dose-rate photon exposures will likely result in irreparable DNA damage with biological consequence. In contrast, delayed and sublethal photon exposures that deliver very low-dose-rates may result in DNA damage that is rapidly repaired [10]. In contrast to photons, neutrons have a high relative biological effectiveness due to increased ionization density leading to clustered DNA damage that is more difficult for the cellular machinery to repair. Even at small doses, neutron exposure can lead to extensive physiological injury [10,11,12]. Further, an urban environment is likely to result in minimal to moderate shielding resulting in total or partial body radiation exposures across dose rates, types, and geometries. The myriad of factors to consider in the development of precise and accurate biomarkers of radiation injury, even while omitting extrinsic determinants, such as physical damage, are extensive. 

The primary objective of this review is to identify the most abundant metabolomic and lipidomic perturbations that are observed in studies with radiation exposure, including animal models of ARS and/or DEARE. Several animal and human studies were reviewed from approximately 65 manuscripts published in peer-reviewed journals. The ten most referenced metabolites were evaluated for directional trends. Those that show agreement across several studies were further evaluated for time- and dose-dependent responses that may indicate suitability for use as diagnostic or prognostic biomarkers. The identification of radiation specific predictive and pharmacodynamic metabolite biomarkers requires global profiling studies with the objective of exploring short and long term timepoints and the use of therapeutics. Both can be better understood after radiation induced diagnostic and prognostic biomarkers are well established. Lipid biomarker candidates were less represented in the manuscripts reviewed; however, several were detected in two or more studies. Trends were not always described, as the focus was sometimes on the lipid detection and structure. 

## 2. Results

### 2.1. Metabolomic Biomarker Candidates

Metabolites most frequently detected and validated in both animal and patient studies were citrulline (C_6_H_13_N_3_O_3_), citric acid (C_6_H_8_O_7_), creatine (C_4_H_9_N_3_O_2_), taurine (C_2_H_7_NO_3_S), carnitine (C_7_H_15_NO_3_), xanthine (C_5_H_4_N_4_O_2_), creatinine (C_4_H_7_N_3_O), hypoxanthine (C_5_H_4_N_4_O), uric acid (C_5_H_4_N_4_O_3_), and threonine (C_4_H_9_NO_3_). General study parameters of each metabolite have been outlined in Table 1. Other metabolites that were moderately abundant among several publications include 2′deoxyuridine (C_9_H_12_N_2_O_5_), arginine (C_6_H_14_N_4_O_2_), palmitic acid (C_16_H_32_O_2_), glycine (C_2_H_5_NO_2_), uridine (C_9_H_12_N_2_O_6_), inositol (C_6_H_12_O_6_), lactic acid (C_3_H_6_O_3_), leucine (C_6_H_13_NO_2_), linoleic acid (C_18_H_32_O_2_), methionine (C_5_H_11_NO_2_S), glutamine (C_5_H_10_N_2_O_3_), hippuric acid (C_9_H_9_NO_3_), tyrosine (C_9_H_11_NO_3_), and sebacic acid (C_10_H_18_O_4_) [4,13,14,15,16,17,18,19,20,21,22,23,24,25,26,27,28,29,30,31,32,33,34,35,36,37,38,39,40,41,42].

### 2.2. Lipidomic Biomarker Candidates

Lipids were less represented in the selection of studies; however, a large selection of lipid classes have shown perturbations when comparing sham or pre- and post-IR specimens. Phosphatidylcholines (PCs), lysophosphatidylcholines (LPCs), triacylglycerides (TGs), sphingomyelins (SMs), phosphotidylinositols (PIs), diacylglycerides (DGs), and cholesteryl esters (CEs) were detected in several lipid studies, however class structures were often variable. The increase of sphingolipid ceramide was noted in a number of studies [7,60,61,62,63]. The most common lipid structure found was a decrease in PC (36:1) from mouse, non-human primates (NHP), and human studies [20,26,60,64]. Additional PCs and TGs that were detected in at least two different studies are: PC (34:0), PC (34:2), PC (36:2), PC (36:5), TG (58:7), TG (52:3), TG (56:6), TG (56:8), TG (58:3), and TG (58:4) [20,26,29,60,64,65].

Trends in lipid classes such as DGs, TGs, and LPCs were observed in two studies using TBI in animal models. Pannkuk et al., 2017c, exposed NHPs to 6.5 Gy of Cobalt-60 gamma source, predicted to induce lethality in 50% of animals over 60 days [66]. Laiaikis et al., 2019a, exposed mice to a mixed field sublethal dose of 3 Gy (equitoxic dose based on mixed field exposures: gamma and neutron). The most robust responses came from the intermediate ratios that contained neutron fractions of 15 or 25%. In contrast, neutron fractions of 5 and 83% showed far fewer statistically significant ion variations compared to control [12]. Table 2 shows lipid trends that agree between the two studies shaded. A decrease in DGs was observed one day post-IR, while increases in TGs and LPCs were observed from approximately 2 to 7 days post-IR [12,66]. Variability may stem from differences in the animal models, IR dose, or radiation type. Pannkuk et al., 2017c, noted that a biphasic response that occurred among metabolites in later time points (21–28 days) may be due to immunological bacterial responses [66].

## 3. Discussion

### 3.1. Acute Radiation Syndrome and Delayed Effects of Acute Radiation Exposure

IR exposure results in a cascade of biological events that is both time and dose dependent [52,67,68]. ARS manifests in four stages—the prodromal syndrome, the latent period, the manifestation of illness, and either death or recovery. The onset, duration, and severity of each phase varies depending on the dose of exposure and cell turnover kinetics of the involved tissue. The prodromal syndrome manifests within minutes to hours and may include early symptoms such as nausea, diarrhea, fatigue, or fever depending on the severity of exposure [67,68,69]. Symptoms clear during the latent period, which may be absent altogether at supralethal doses of exposure, followed by the manifestation of illness that is classically associated with three subsyndromes. These are the hematopoietic-subsyndrome (~0.5–5 Gy), the gastrointestinal subsyndrome (~5–10 Gy), and the cardiovascular or central nervous systems associated with extremely high doses of up to 100 Gy, and death within approximately 48 h [67,68,69]. Low linear-energy transfer radiation (e.g., photons) induces damage primarily through the radiolytic hydrolysis of water resulting in damage to cellular macromolecules [70,71]. In contrast, high linear energy transfer (e.g., neutrons) exacts damage through clustered DNA damage primarily characterized by double strand breaks and non-double strand break oxidative clustered DNA lesions [72]. Downstream of the initial ionizing event and subsequent biological cascade of free radical species, lipid molecules of cellular membrane are prime targets of degradation resulting lipid peroxidation [73,74]. Lipid hydroperoxides have been shown to significantly increase in patients receiving TBI treatment within 10 days, while patients receiving chemotherapy alone showed no change [75].

DEARE occurs weeks to months post-exposure. Late toxicity develops with the release of cytokines and growth factors, and perturbations in the tissue microenvironment (e.g., vascular damage, tissue hypoxia). Although far less understood, it is believed that the long-term effects of an excessive pro-inflammatory response coupled with immune dysregulation, and the generation of reactive oxygen/nitrogen oxide species (RNOS) leads to organ dysfunction as well [67,70,71]. These are believed to be the catalysts to necrosis, fibrosis, and slow wound healing; reflecting the impaired regeneration of injured tissue due to prolonged damage of cell fractions [76]. Chronic lung injury (e.g., radiation pneumonitis/fibrosis) presents as pulmonary fibrosis, pneumonitis, pleural effusion, edema, and vascular leakage [77]. Chronic fibrosis takes months to years to develop, while pneumonitis manifests in 2 to 6 months post exposure [1,2,28]. Pulmonary fibrosis among other chronic injuries lead to poor long-term quality of life or even death [69,77]. Currently there are no diagnostic biomarkers or effective treatments for radiation induced lung injury [28,78].

### 3.2. Metabolomic Biomarker Discovery and Evaluation

A multi-parametric approach is more likely to predict the radiation dose received and perhaps even the tissue affected when coupled with the duration of time from exposure. While system biology includes many approaches to biomarker discovery, metabolomics appears to have multiple promising characteristics. Metabolites are defined as small molecules less than approximately 1 kDa. The use of metabolomic biomarkers will reflect the real-time physiological status that results from a cascade of cellular processes. The radiological insult triggers an epigenomic event followed by genomic, proteomic, and transcriptomic responses. The metabolomic changes resulting from the cascade of events reflect a real-time phenotypic and functional response [7].

The search for radiation induced metabolomic changes has utilized mass-spectrometry (MS) and nuclear magnetic resonance (NMR) platforms in which endogenous molecules of variable physical and chemical properties can be detected, validated, and quantified.

The first phase in biomarker discovery is an untargeted screening approach that can identify hundreds of candidates when comparing metabolic signatures of sham or pre- and post-irradiation samples [5,79]. This global profiling phase produces massive data sets that require extensive computational methods for analysis. Analysis of differential metabolite expression will be performed between sham or pre- versus post-IR animal cohorts [5]. Metabolites identified require validation using external references [80]. Several significantly perturbed metabolites have been identified from profiling metabolites from biofluids, all of which may provide appropriate candidates for diagnostic biomarkers depending on the agreement among different animal models.

The second phase is a targeted approach that utilizes information gathered from the first phase to evaluate and analyze trends [5,7]. Like other platforms, quantitative measures of metabolites are performed with calibration curves and using normalization techniques [5]. Metabolomic trends may include the direction of perturbation, and expand into a time- and dose-dependent response. Significant trends may determine which metabolites are appropriate for prognostic biomarkers depending on the agreement among different animal models. Numerous studies have utilized both phases while evaluating different parameters such as species, radiation dose, and sampling timepoints. This review has identified metabolomic biomarkers that are detected and validated most frequently in current literature.

Citric acid, creatine, citrulline and uric acid exhibit largely consistent trends, with 70% or more agreement among the studies reviewed. In contrast, taurine, carnitine, xanthine, creatinine, hypoxanthine, and threonine show variable trends among the studies (67% study trend agreement or less). This indicates that multiple factors may define the metabolic response. A more defined and systematic review may elucidate whether trends are biphasic due to differences in IR dose, sampling time, analytical platform, or species. Further, factors that are not encompassed in this review and could produce variability are sex, age, diet composition, the level of food and water intake, and especially in human studies, ethnicity, prescription use, and health conditions, etc., prior to IR. Animal studies have compared metabolic responses in male versus female cohorts, with significant differences reported between the two [20,52]. Physiological metabolic differences between the sexes leads to dimorphic responses in injury or disease. In addition to hormones, genes and post-translational changes differ between men and women, indicating unique enzymatic activity and cellular signaling responses may occur between the sexes as well [81]. Because citric acid, creatine, citrulline and uric acid show 70% or more agreement in directional trends from several studies, they were further evaluated for use as ubiquitous diagnostic or prognostic biomarkers.

### 3.3. Citric Acid

A decrease in citric acid was observed in 70% of the studies reviewed. Chen et al., 2011, Goudarzi et al., 2014a, 2014b, 2015c, Lanz et al., 2009, Pannkuk et al., 2017a, and 2019a observed decreases post-IR in mouse, rat, and NHP models [22,24,33,34,40,41,42]. In contrast, three out of ten studies observed an increase in citric acid. Tyburski et al., 2008, did not show a significant increase in mouse urine at one day post exposure [43]. Goudarzi et al., 2016a, and Wibom et al., 2010, measured significant increases in sample matrices such as feces and brain tissue [39,44]. Most citric acid decreases were observed during early timepoints (within 5 days post-IR) [40,43], with levels returning to baseline by day 7 [42]. In contrast, internal exposure using Cesium-137 or Strontium-90 showed decreased levels in urine persisted, only returning to baseline levels by day 30 post-IR [24,41]. Citric acid may be an appropriate diagnostic biomarker early after exposure (within 7 days) depending on the IR exposure type. With more research the metabolite may serve as a prognostic biomarker because of both time and dose dependent trends.

Citric acid is an intermediate of the tricarboxylic acid (TCA) cycle [82]. A reduction in TCA intermediates such as citric acid indicates mitochondrial dysfunction [34,83]. The mechanism of action for decreasing citric acid post-IR may involve the oxidative metabolism pathway in response to reactive oxygen species (ROS). Radiation injury generates RNOS and reactive oxygen intermediates such as hydrogen peroxide (H_2_O_2_) [67,84,85]. A decrease in citric acid post-IR in the presence of oxidative stress may involve the enzyme aconitase, responsible for reversibly converting citric acid to isocitric acid, and the oxidative metabolism pathway [83]. Mitochondrial aconitase (ACO2) contains a cubane cluster active site [4Fe-4S]^2+^ required for enzymatic activity, and very susceptible to oxidative stress. Further, the cytosolic iron regulatory protein-1 (IRP-1) contains a similar cubane cluster active site [4Fe-4S] with comparable functions and susceptibilities to oxidative stress [83]. The induction of IRP-1 by H_2_O_2_ leads to the binding of iron response elements (IREs), the destruction of the cubane cluster, and a loss in enzymatic function [84]. Citric acid also interacts with the IRP-1 cubane cluster, resulting in the inhibition of IRE binding. Pantopoulos et al., 1995, used B6 fibroblasts to show that citric acid inhibits the binding of the IRP-1 and IRE complex in untreated cells, however failed to inhibit the binding in the presence of H_2_O_2_ [84]. In summary, the mechanism of a radiation induced decrease in citric acid may result from the susceptibility of ACO2, the enzyme responsible for converting citric acid to isocitric acid within the TCA cycle, to oxidative stress.

### 3.4. Creatine

An increase in creatine was observed in 86% of the studies reviewed. Chen et al., 2011, 2018, Mak et al., 2015a, Johnson et al., 2012, Pannkuk et al., 2018a and 2019b, show agreement in mouse, rat, and NHP models [8,18,42,45,46,48]. Mak et al., 2015a, showed a strong dose and time-dependent response in rats up to 72 h post-IR. Creatine levels were normalized to creatinine [45]. Studies listed in Table 1 show a similar trend with levels correlating to IR dose. Pannkuk et al. 2015, showed a small but significant drop in creatine levels at 2 Gy, followed by a dose-responsive increase up to 10 Gy [47]. Creatine levels have been shown to significantly increase within 7 days post-IR, however data does not show extended perturbations with time. Creatine may be a candidate for a diagnostic biomarker, and perhaps even prognostic if further research correlates early measures with survival.

Creatine is a metabolite that is gained through food intake, and synthesized from arginine, methionine and glycine [42]. It is essential to healthy cell and tissue function and is utilized by the amino acid pathway [86]. Elevated levels of creatine in urine are likely due to the inability of injured muscle to take up and use the available metabolite [18,42,45,87]. IR induced myopathy has been observed in human and animal studies, however the causative mechanism has yet to be elucidated [87,88,89]. Monohydrate creatine supplementation has been tested in patients with variable myopathies, however results were mixed [88]. Ghosh et al., 2015, measured serum creatine kinase, an intermediate to creatine uptake, levels in adult cancer survivors with radiation-induced chronic myopathy; however, values were normal or minimally elevated [89]. More research is needed to determine whether intermediates to creatine uptake such as skeletal muscle creatine transporter protein, or sarcomeric mitochondrial creatine kinase may be instrumental in myopathy [88,90].

### 3.5. Citrulline

A decrease in citrulline has been observed in 87% of the studies reviewed. Jones et al., 2014a, 2014b, 2015a, 2015b, 2019a, 2019b, Kurland et al., 2015, Tang et al., 2013, Shim et al., 2014, Pannkuk et al., 2016b, and Onal et al., 2011, show agreement in mouse, rat, pig, NHP, and human models [5,19,20,31,35,49,50,52,53,54,55]. Citrulline has been used as a biomarker in radiation research for over a decade, with present day studies utilizing measures to score gastrointestinal (GI) injury in animals post-IR [6,35,91,92]. Studies have shown a dose and time-dependent reduction of blood and urine derived citrulline post IR exposure [5,19,49,50]. Significant correlations between the decrease in circulating citrulline and the decrease in the number of crypts in the small intestines have been shown, linking the biomarker with a pathological effect of radiation [49,50]. As Table 1 shows, agreement across species, radiation doses, and analytical platforms indicate similar decreasing measures from circulating citrulline. The time points of sampling agree extensively as well, with citrulline measures decreasing until about day 7 post-IR, and then gradually returning to baseline upon recovery. In contrast, Goudarzi et al., 2016b, used Strontium-90 (internal 2 Gy dose), Cesium 137 (internal 4.1 Gy dose, or X-ray (external 4.4 Gy dose)) to evaluate the injury from variable sources of irradiation. While both internal exposure methods measured a decrease in urine derived citrulline levels, the X-ray samples measured an increase on day 5 of sampling [51]. Laiakis et al., 2019b, also showed an increase in citrulline day 1 post-IR. Levels returned to baseline by day 3, however gradually increased again from day 21 to 60 [17]. This study measured metabolites from NHP saliva, in contrast to blood or tissue derived measures used in other studies. This indicates that more research may be required to determine the variability of measuring metabolites based on the sampling matrix.

Animal data showing decreases in citrulline levels return to baseline within approximately 7 days of exposure. However, samples of patients treated with pelvic radiotherapy (45–75.6 Gy) were measuring significantly lower levels of citrulline for as long as 4 months post-IR [55]. These measures reflect local damage, indicating that citrulline may be appropriate for measuring organ specific injury. However, Jones et al., 2019b, compared organ specific damage (jejunum) to circulating citrulline levels and noted that additional biomarkers would better determine GI specific damage [35]. A dose and time-dependent response indicates this metabolite may serve as a diagnostic and even prognostic biomarker for acute and chronic injury. However, Jones et al., 2019b, also indicated plasma citrulline is not appropriate for predicting survival [35].

Citrulline has been identified as an intermediate in the urea cycle, as well as an end product of the nitrogen glutamine metabolism [93]. Small bowel enterocytes are responsible for a vast majority citrulline released into circulation by means of the glutamine metabolism pathway [94]. The kidney is one of the main tissues to utilize the metabolite in a healthy physiological state [93]. A decrease in circulating citrulline has been correlated with the other intestinal specific measures post-IR, such as small bowel epithelial cell loss [93].

### 3.6. Uric Acid

An increase in uric acid was observed in 78% of the studies reviewed. Laiakis et al., 2012, 2014a, Lee et al., 2012, Goudarzi et al., 2014b, 2015b, Mak et al., 2015a, and Johnson et al., 2012, show agreement in mouse, rat, and NHP models [23,36,41,45,46,56,58]. Increases were also observed in human patients after radiotherapy [58]. Laiakis et al., 2017a, is an exception in which uric acid levels were significantly decreased in mouse serum at days 1 and 7 post exposure. Neutron exposure was utilized, indicating that the type of radiation may induce variable metabolite changes when compared to x-ray or gamma [4]. Roszkowski et al., 2008, observed significant decrease in human plasma uric acid levels from patients treated with radiotherapy [59]. Many studies captured an early increase of the metabolite (within days). In contrast, Goudarzi et al., 2014b and 2015b, showed internal exposure using Cesium-137 resulted in a prolonged increase in levels until day 20 post exposure. Levels returned to baseline by day 30 [23,41]. A similar prolonged detection of citric acid was observed after internal exposure to Strontium-90 [24].

**Figure 1:**
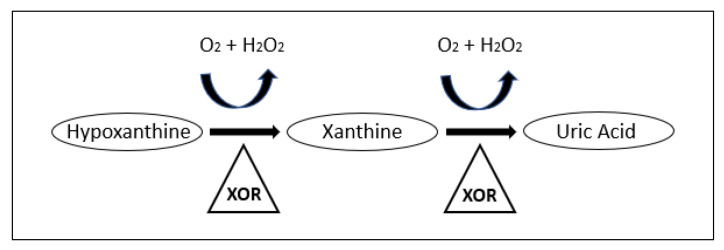
Purine metabolism pathway leading to the production of Uric acid [97]. Reproduced with permission from [L.E. Scheepers et al.], [THU0519 Xanthine Oxidase Gene Variants and Their Association with Blood Pressure and Incident Hypertension: A Population Study]; published by [BMJ Publishing Group Ltd], [2016].

Uric acid is a free radical scavenger that can protect DNA in the event of damage. The metabolite is a product of purine metabolism in which xanthine oxidoreductase (XOR) breaks down hypoxanthine and xanthine. The oxidation is thought to result in the clearing of free oxygen radicals and the formation of uric acid [59,95,96]. A simple purine metabolism pathway is shown in Figure 1. Xanthine and hypoxanthine were also found to be increased in several studies as shown in Table 1, indicating that increases in the purine metabolism pathway may be ubiquitous.

Uric acid is a potent anti-oxidant that may serve well as a diagnostic biomarker. The metabolite has been used as a prognostic biomarker for different diseases. For example, Yang et al., 2019, showed the metabolite to be a significant blood derived prognostic biomarker for overall survival in patients with advanced gastric cancer [95]. Paithankar et al., 2020, utilized in-vivo and in-vitro experiments to analyze changes in uric acid levels post-IR. Drosophila flies, which are normally radio-resistant, became susceptible to radiation (1000 Gy) after treatment with Allopurinol, a drug that inhibits xanthine oxidase and reduces overall uric acid levels. Further, human dermal fibroblast cells were supplemented with uric acid post-IR (2 Gy), resulting in increased survival when compared to non-treated controls [97]. An increase in uric acid may have a protective effect post-IR, and may serve as a promising therapeutic in higher organisms as well.

### 3.7. Lipidomics

Lipidomics is a large subset of metabolomics that encompasses the biochemical characterization of lipids within an organism, tissue, or cell. Human plasma contains thousands of lipid species, providing a rich environment to detect perturbations in sham or pre- and post-radiation exposure specimens [98]. Glycerophospholipids, or phospholipids (PLs), are most abundant; composing biological membranes and are fundamentally involved in cellular processes. PCs and other PL derivatives such as LPCs are signaling molecules that take part in regulating cellular apoptosis and proliferation [60]. Several blood derived measurements of lipids including PCs, LPCs, TGs, SMs, and CEs have shown perturbations when comparing sham or pre- and post-irradiation specimens.

RNOS will react extensively with lipid molecules, resulting in lipid degradation, and inducing both direct and indirect inflammatory responses [60,85,99]. Acute perturbations in specific lipid concentrations results during early timepoints post-IR [60]. The quantification of lipid species in radiation therapy treated patients revealed that radiation induced serum lipidome perturbations were resolved within 1–2 months post-IR treatment [60]. In contrast, serum proteome perturbations were still detected several months post-IR treatment [60]. This suggests that radiation induced perturbations in the serum lipidome may reflect changes within earlier timepoints, and possibly different injury pathways when compared to the proteomic responses [60]. However, more long-term studies may indicate that lipid measures are appropriate biomarkers for DEARE as well [66].

The structural diversity of the mammalian lipidome is so vast that it requires systematic nomenclature for organized and clear classifications [79,100]. Metabolomic nomenclature is first listed by class, such as PC. Analytical platforms like mass spectrometry are sensitive enough to identify lipid subclasses with the sum composition, or the total number of carbon in the fatty acid chain and the number of double bonds (ratio) [79]. For example, PC (36:2) indicates the lipid structure contains 36 total carbon with 2 double bonds [52]. However, a complete structurally defined molecular lipid will provide more information with respect to the regiochemistry, unsaturation, or stereochemistry [79]. The studies evaluated in this review included lipids classified by sum composition only. While several lipid classes have shown perturbations when comparing sham or pre- versus post-IR samples, the variable sums and ratios within each metabolite class accumulate several distinct structures.

### 3.8. Ceramide

Ceramide is a sphingolipid that has been recognized in a number of irradiation studies including Jones et al., 2017a, Jelonek et al., 2014, Lin et al., 2000, and Kolesnick et al., 2003 [60,61,62,63]. Jones et al., 2017a, showed that ceramide increased in mouse lung tissue within 24 h post-IR [63]. Studies have shown that radiation induces sphingomyelin hydrolysis to ceramide in several cell types. Ceramide then acts as a messenger to initiate apoptosis in endothelial, lymphoid, and hematopoietic cells [61,62,101]. Anti-ceramide antibody treatment has shown to prevent radiation gastrointestinal syndrome in mice exposed to 10–15 Gy (TBI) when compared to non-treated controls. A therapeutic dose-response was observed when compared to the number of surviving small intestinal crypts. Ninety-day survival increased from 0% to 80% in antibody treated animals, however treatment was administered 15 min pre-IR [102]. Jones et al., 2017b, used the drug BIO 300 to test mitigation in radiation induced pulmonary damage and dysfunction in the mouse model. BIO 300 is a formulation of synthetic genistein that has demonstrated efficacy in preventing lung injury. While a direct measure of ceramide was not made, sphingolipids such as SMs correlated with the efficacy of treatment. A radiation induced decrease in SMs is consistent with an increase in activity of acid sphingomyelinase, the enzyme that metabolizes SM into ceramide. The increase in ceramide then induces apoptotic signaling. BIO 300 treatment significantly increased the levels of four SM class structures including SM 16:0, 24:0, (OH)16:1, and (OH)24:1, and may have contributed to the biphasic reduction of ceramide induced apoptosis [28]. More research is required to determine whether ceramide would serve as a therapeutic target, however, initial data is promising.

### 3.9. Tissue Specific Biomarkers

With the exception of citrulline, organ specific injury was under-represented in this review, which is primarily composed of TBI animal study models. Studies using whole thorax lung irradiation (WTLI) to induce radiation induced lung injury (RILI) measured metabolites from biopsied lung tissue or serum [28,64,78,103]. Although significant differences were detected in metabolomic signatures between sham or pre- and post-IR cohorts, little agreement was found among studies. Significant perturbations occurred in SMs [28], PCs [64], and PIs [78]; however, lipids and structures did not overlap in detection among studies.

### 3.10. Creatinine Use in Normalization Techniques

Creatinine is an abundant metabolite sometimes used to normalize other metabolite measures in urine and serum. It is also used as an indicator of kidney function by measuring kidney glomerular filtration rate (GFR), and an indicator of kidney damage. Urine metabolites are normalized using creatinine levels to counterbalance fluid intake levels [8,104,105]. The metabolite has also been a trusted normalizing factor in serum because of creatinine homeostasis [8]. However, as Table 1 shows, trends in creatinine levels vary significantly post-IR in mouse, NHP, and human studies. Jones et al., 2019a, and Moren et al., 2016, found significant decreases in creatinine serum levels [21,52]. Chen et al., 2018, Johnson et al., 2012, and Pannkuk et al., 2015, observed significant differences in NHP creatinine urine levels when comparing sham or pre- to post-IR samples [8,46,47]. A decrease in creatinine is observed in 67% of the studies reviewed, and the perturbations observed were significant in 5 out of the 6 studies listed. Normalizing data to a single metabolite that has been shown to significantly change in several studies could mask true values. Alternative normalization techniques that encompass a more complete evaluation of sample concentration includes osmolality, or ‘total useful signal’ using the MS platform to calculate the sum of features that are common to all samples measured [105].

## 4. Materials and Methods

Approximately 65 manuscripts published in the peer-reviewed journals were screened for metabolite and lipid changes observed when comparing sham or pre- to post-IR measures. The lipid studies evaluated for this review included those sub-classified by sum composition only. Metabolites and lipids (hereinafter both will be referred to as metabolite) were listed individually and linked to source information. Without counting duplicate listings, approximately 390 variable metabolites were observed. Once the review of all manuscripts was completed the list was sorted alphabetically, pooling all duplicate metabolites from different studies. Those that were listed most, ranging from 6 studies (creatinine) to 15 studies (citrulline) were included in Table 1. General study parameters such as species, radiation geometry, dose (Gy), radiation type, timepoints post-IR, sample matrix, analytical platform, profiling or targeted study technique, significance, and directional trends were outlined. The ten metabolites listed in Table 1 are from 43 manuscripts. Other metabolites listed by 4–5 manuscripts were described in the body of the results section; however, were not included in the summary table.

Metabolites were then evaluated for agreement in directional trends. Single directional trends such as an increase or decrease in metabolites post-IR were recorded as increase (Inc) or decrease (Dec) respectively. Bi-directional trends were recorded according to what was observed by time. For example, ‘Inc → Dec’ indicates an increase in metabolite concentration occurred, followed by a decrease post-IR. In contrast, trends showing multiple bi-directional trends within sampling timepoints, or trends that disagree by dose response are listed as ‘variable’ (V). An arbitrary 70% agreement in trend was utilized to determine which metabolites would be described further, evaluated for time and dose dependent responses, and possibly serve as diagnostic or prognostic biomarkers. The percent agreement for the directional trend of each metabolite is as follows: citrulline (87% study agreement (SA): decrease trend (DT)), creatine (86% SA: increase trend (IT)), uric acid (78% SA: IT), citric acid (70% SA: DT), carnitine (63% SA: IT), xanthine (63% SA: IT), taurine (64% SA: IT), creatinine (67% SA: DT), hypoxanthine (57% SA: IT), and threonine (57% SA: IT).

## 5. Conclusions

The ideal emergency response plan to a mass nuclear event includes early phase testing (within one week) using a panel of biomarkers that would guide individualized and targeted treatment. Extensive progress has been made in screening for metabolite perturbation post-IR using mass spectrometry platforms, animal models, and occasionally data collected from radiation-treated cancer patients. Several studies have elucidated significant metabolomic changes and even trends when comparing sham or pre- versus post-IR samples. Metabolites most frequently detected and validated post-IR preclinical studies using animal models of ARS and DEARE were citrulline, citric acid, creatine, taurine, carnitine, xanthine, creatinine, hypoxanthine, uric acid, and threonine. Studies show largely consistent trends in citric acid (70% of studies reviewed), creatine (increase in 86% of studies reviewed), citrulline (decrease in 87% of studies reviewed), and uric acid (increase in 78% of studies reviewed). Citric acid, creatine, citrulline, and uric acid are strong candidates for diagnostic biomarkers due to time- and dose-dependent measures. Although less represented, lipids such as PC (36:1) and sphingolipid ceramide showed consistent trends from a number of studies. Research shows ceramide may be an appropriate therapeutic target. Further studies and analysis will elucidate which metabolites and lipids are appropriate candidates for prognostic, predictive, and even pharmacodynamic biomarkers. Further, some may be appropriate for use as prognostic biomarkers to chronic IR induced injury with continued research outlining long term disease outcomes.

## Figures and Tables

**Table 1:** Metabolites most frequently detected in review of several animal and human studies.

Metabolite	Species	Radiation Geometry	Dose (Gy)	Radiation Type	Timepoint(s) Post-IR	Sample Matrix	Analytical Platform	Technique	Source	Significance	Trend
Citric Acid	Mouse	TBI	3, 8	γ-ray	1 day	Urine	LC–MS	P	Tyburski et al. 2008 [43]	NS	Inc
	.	.	8	x-ray	1–7 days	.	^1^H NMR	.	Chen et al. 2011 [42]	*	Dec
	.	.	1.1,4.45	γ-ray	2–5 days	.	LC–MS	.	Goudarzi et al. 2014a [40]	*	Dec
	.	Internal	1.95–9.91	.	2, 5, 20, 30 days	.	.	.	Goudarzi et al. 2014b [41]	*	Dec
	.	.	1.81–5.25	β	7–30 days	.	.	.	Goudarzi et al. 2015c [24]	NS	Dec
	.	TBI	5,12	x-ray	3, 14, 30 days	Feces	.	.	Goudarzi et al. 2016a [44]	*	Inc
	Rat	.	3	γ-ray	1, 2, 3 days	Urine	GC–MS	.	Lanz et al. 2009 [33]	*	Dec
	NHP	.	2, 4, 6, 7, 10	.	7 days	.	.	.	Pannkuk et al. 2017a [22]	*	Dec
	.	.	4	.	1–60 days	Urine + Serum	.	.	Pannkuk et al. 2019a [34]	*	Dec
	Human	PBI	2–3.4	x-ray	36 h	Tissue (brain)	.	.	Wibom et al. 2010 [39]	*	Inc
Creatine	Mouse	TBI	8	x-ray	1–7 days	Urine	^1^ H NMR	P	Chen et al. 2011 [42]	*	Inc
	Rat	.	0.5–10	γ-ray	6–72 h	.	LC–MS	P + T	Mak et al. 2015a [45]	*	Inc
	NHP	.	6.5, 7.2	.	12, 24 h	Serum	.	T	Pannkuk et al. 2018a [18]	NS	Inc
	.	.	1, 3.5, 6.5, 8.5	.	12–72 h	Urine	.	P + T	Johnson et al. 2012 [46]	*	Inc
	.	.	2, 4, 6, 7, 10	.	7 days	.	DMS–MS	T	Chen et al. 2018 [8]	U	Inc
	.	.	2, 4, 6, 7, 10	.	7 days	.	LC–MS	P	Pannkuk et al. 2015 [47]	*	Dec → Inc
	.	.	4	.	1–60 days	Urine + Serum	.	P + T	Pannkuk et al. 2019b [48]	**	Inc
Citrulline	Mouse	TBI	8–15	x-ray	1–6 days	Plasma	LC–MS	T	Jones et al. 2014a [5]	*	Dec
	.	TBI/PBI	10, 50	γ-ray	1 day	.	GC/LC–MS	P + T	Kurland et al. 2015 [31]	NS	Dec
	.	TBI	6–15	x-ray	1–6 days	.	LC–MS	T	Jones et al. 2015a [49]	**	Dec
	.	.	13	.	1–7 days	.	.	.	Jones et al. 2014b [50]	U	Dec
	.	Internal/TBI	2, 4.1, 4.4	β/γ-/x-ray	Up to 30 days	Urine	.	.	Goudarzi et al. 2016b [51]	**	V
	.	TBI	8, 8.72	γ-ray	3, 9 days	Serum	.	P	Jones et al. 2019a [52]	**	Dec
	.	.	8, 10, 12, 14	x-ray	1, 3 days	Jejunum + Plasma	.	T	Jones et al. 2019b [35]	**	Dec
	Rat	.	2, 4, 6, 8	.	5, 24, 72 h	Plasma	.	.	Tang et al. 2013 [19]	*	Dec
	Pig	.	10	γ-ray	1–7 days	.	.	.	Jones et al. 2014b [50]	U	Dec
	.	PBI	10, 15	.	Up to 37 days	.	.	.	Shim et al. 2014 [53]	*	Dec
	NHP	TBI	2, 4, 6, 7, 10	.	7 days	Serum	.	.	Pannkuk et al. 2016b [20]	*	Dec
	.	.	4	.	1–60 days	Saliva	.	P	Laiakis et al. 2019b [17]	**	V
	.	TBI/PBI	7.5–13	x-ray	Up to 180 days	Plasma	.	T	Jones et al. 2015b [54]	**	Dec
	.	TBI	10.5	.	1–7 days	.	.	.	Jones et al. 2014b [50]	U	Dec
	Human	PBI	45–75.6	.	21–112 days	.	.	.	Onal et al. 2011 [55]	**	Dec
Taurine	Mouse	TBI	3, 8	γ-ray	1 day	Urine	LC–MS	P	Tyburski et al. 2008 [43]	**	Inc
	.	.	8	x-ray	1–7 days	.	^1^H NMR	.	Chen et al. 2011 [42]	*	Inc → Dec
	.	.	3, 8, 15	γ-ray	Up to 15 days	.	LC–MS	.	Laiakis et al. 2012 [56]	*	Inc
	.	Internal	1.95–9.91	.	2–30 days	.	.	.	Goudarzi et al. 2014b [41]	*	Inc
	.	.	1.95	.	2–30 days	Serum	.	.	Goudarzi et al. 2015b [23]	**	Dec
	.	TBI	5, 12	x-ray	3 days	Feces	.	.	Goudarzi et al. 2016a [44]	*	Inc
	.	.	0.25, 1, 4	Neutron	1, 7 days	Urine + Serum	.	.	Laiakis et al. 2017a [4]	*	Dec
	Rat	.	3	γ-ray	Up to 3 days	Urine	.	.	Johnson et al. 2011 [13]	**	Inc
	NHP	.	2, 4, 6, 7, 10	.	7 days	.	.	.	Pannkuk et al. 2016a [29]	*	Inc
	.	.	2, 4, 6, 7, 10	.	7 days	Serum	.	.	Pannkuk et al. 2015 [47]	*	Dec → Inc
	.	.	1, 3.5, 6.5, 8.5	.	12–72 h	Urine	.	P + T	Johnson et al. 2012 [46]	*	Inc
Carnitine	Mouse	TBI	8.0, 8.72	γ-ray	3, 6, 9 days	Serum	LC-MS	P	Jones et al. 2019a [52]	*	Dec
	.	Internal	1.95–9.91	.	2–30 days	.	.	.	Goudarzi et al. 2015b [23]	*	Dec
	.	TBI	8	.	1 day	.	.	.	Laiakis et al. 2014b [57]	*	Inc
	Rat	.	0.5–10	.	6,24,48,72 h	Urine + Serum	.	P + T	Mak et al. 2015a [45]	*	Inc
	NHP	.	6.5	.	12, 24 h	Serum	.	T	Pannkuk et al. 2018a [18]	**	Dec → Inc
	.	.	2, 4, 6, 7, 10	.	7 days	Urine	.	P	Pannkuk et al. 2015 [47]	**	Inc
	.	.	2, 4, 6, 7, 10	.	7 days	Serum	.	T	Pannkuk et al. 2016b [20]	**	Inc
	.	.	4	.	1–60 days	Urine + Serum	.	P + T	Pannkuk et al. 2019b [48]	**	Inc
Xanthine	Mouse	TBI	1, 2, 3	γ-ray	Up to 9 days	Urine	LC–MS	P	Tyburski et al. 2009 [14]	*	Inc
	NHP	.	4	.	1–60 days	Urine + Serum	.	P + T	Pannkuk et al. 2019b [48]	*	V
	.	.	4	.	1–60 days	Saliva	.	P	Laiakis et al. 2019b [17]	*	V
	.	.	6.5	.	12, 24 h	Serum	.	T	Pannkuk et al. 2018a [18]	*	Inc
	.	.	2, 4, 6, 7, 10	.	7 days	Urine	DMS–MS	.	Chen et al. 2018 [8]	NS	Inc
	.	.	1, 3.5, 6.5, 8.5	.	12–72 h	.	LC–MS	P + T	Johnson et al. 2012 [46]	*	Inc
	.	.	2, 4, 6, 7, 10	.	7 days	.	.	P	Pannkuk et al. 2015 [47]	NS	V
	Human	.	3.75	x-ray	4–24 h	.	.	.	Laiakis et al. 2014a [58]	*	Inc
Creatinine	Mouse	TBI	8.0, 8.72	γ-ray	3, 6, 9 days	Serum	LC–MS	P	Jones et al. 2019a [52]	*	Dec
	.	.	2	.	2 months	Intestinal issue	.	.	Cheema et al. 2014 [30]	*	Inc
	NHP	.	2, 4, 6, 7, 10	.	7 days	Urine	DMS–MS	T	Chen et al. 2018 [8]	NS	Dec
	.	.	1, 3.5, 6.5, 8.5	.	12–72 h	.	LC–MS	P + T	Johnson et al. 2012 [46]	*	Inc
	.	.	2, 4, 6, 7, 10	.	7 days	.	.	P	Pannkuk et al. 2015 [47]	*	Dec
	.	PBI	2	x-ray	1 day	Serum	GC–MS	.	Moren et al. 2016 [21]	*	Dec
Hypo-xanthine	Mouse	TBI	5, 12	x-ray	3 days	Feces	LC–MS	P	Goudarzi et al. 2016a [44]	*	Inc
	NHP	.	4	γ-ray	1–60 days	Saliva	.	.	Laiakis et al. 2019b [17]	**	Inc
	.	.	1, 3.5, 6.5, 8.5	.	12–72 h	Urine	.	P + T	Johnson et al. 2012 [46]	*	Inc
	.	.	2, 4, 6, 7, 10	.	7 days	.	.	P	Pannkuk et al. 2015 [47]	**	Inc
	.	.	2, 4, 6, 7, 10	.	7 days	.	.	.	Pannkuk et al. 2016a [29]	**	Dec
	.	.	4	.	1–60 days	Urine + Serum	.	P + T	Pannkuk et al. 2019b [48]	**	Dec
	Human	.	3.75	x-ray	4–24 h	Urine	.	P	Laiakis et al. 2014a [58]	**	Dec
Uric Acid	Mouse	TBI	0.25, 1, 4	Neutron	1, 7 days	Urine + Serum	LC–MS	P	Laiakis et al. 2017a [4]	*	Dec
	.	.	10 (cGy)	x-ray	36 h	Plasma	GC–MS	.	Lee et al. 2012 [36]	*	Inc
	.	Internal	1.95–9.91	γ-ray	2–30 days	Urine	LC–MS	.	Goudarzi et al. 2015b [23]	**	Inc
	.	TBI	3, 8, 15	.	Up to 15 days	.	.	.	Laiakis et al. 2012 [56]	*	Inc
	.	Internal	1.95–9.91	.	2–30 days	.	.	.	Goudarzi et al. 2014b [41]	*	Inc
	Rat	TBI	0.5 - 10	.	6–72 h	.	.	P + T	Mak et al. 2015a [45]	*	Inc
	NHP	.	1, 3.5, 6.5, 8.5	.	12–72 h	.	.	P + T	Johnson et al. 2012 [46]	*	Inc
	Human	.	3.75	x-ray	4–24 h	.	.	P	Laiakis et al. 2014a [58]	**	Inc
	.	PBI	70	.	U	Plasma	LC/GC–MS	T	Roszkowski et al. 2008 [59]	**	Dec
Threonine	Mouse	TBI	8, 10, 12, 14	x-ray	1, 3 days	Jejunum + Plasma	LC–MS	T	Jones et al. 2019b [35]	*	Inc	
	.	TBI+PBI	10, 50	γ-ray	1 day	Liver + Plasma	LC/GC–MS	P + T	Kurland et al. 2015 [31]	*	Dec	
	Rat	TBI	2, 4, 6, 8	x-ray	5, 24, 72 h	Plasma	LC–MS	T	Tang et al. 2013 [19]	U	Inc	
	.	.	0.75, 3, 8	γ-ray	1 day	Serum	GC–MS	P	Liu et al. 2013 [27]	*	Inc	
	NHP	.	4	.	1–60 days	Urine + Serum	.	.	Pannkuk et al. 2019a [34]	*	Inc	
	.	.	2, 4, 6, 7, 10	.	7 days	.	.	.	Pannkuk et al. 2017a [22]	*	Dec→ Inc	
	Human	PBI	2 (multiple)	x-ray	1 day	Serum	.	.	Moren et al. 2016 [21]	*	Dec	

Table 1 abbreviations and key: *Species:* NHP (Non-Human Primate). *Radiation Geometry*: TBI (Total Body Irradiation), PBI (Partial Body Irradiation). *Analytical Platform*: LC-MS (liquid chromatography mass spectrometry), ^1^ H NMR (hydrogen-1 nuclear magnetic resonance), GC-MS (gas chromatography mass spectrometry), DMS-MS (Differential mobility spectrometry mass spectrometry). *Technique*: Metabolite identification technique P (global profiling), or T (targeted). *Significance*: * *p*-value ≤ 0.05, ** *p*-value ≤ 0.001 in at least one parameter when comparing sham or pre- versus post-IR, NS (No significance). *Trend*: Inc (increase), Dec (decrease), or V (variable) is comparing sham or pre- to post-IR. *All columns*: U (Unknown). Any spaces designated with a point (.) indicates the parameter is identical to the information provided above.

**Table 2:** A comparison of trends in two lipidomic studies.

Timepoint(s) Post-IR	DG	TG	LPC	Radiation Type	Dose (Gy)	Sample Matrix	Analytical Platform	Technique	Source	Species
**Day 1**	Dec	Dec	Dec	γ-ray	6.5	serum	LC-MS	P	Pannkuk et al., 2017c [66]	NHP
**Day 2–6**	Inc	Inc	*Inc							
**Day 8–12**	Inc	Inc	BL							
**Day 21–28**	Dec	BL	BL							
**Day 1**	* Dec	BL	BL	γ-ray/ neutron	3	serum	LC-MS	P + T	Laiakis et al., 2019a [12]	Mouse
**Day 7**	* Dec	* Inc	* Inc							

Table 2 abbreviations and key: Agreement in lipid changes within a similar timepoint between studies have been shaded. * *p* ≤ 0.05 in at least one lipid subclass. *Trend*: Inc (increase), Dec (decrease), or V (variable). Baseline concentrations (BL) *Analytical Platform*: LC-MS (liquid chromatography mass spectrometry). *Technique*: Metabolite identification technique P (global profiling), or T (targeted). *Species:* NHP (Non-Human Primate).

## References

[1] Van Dyk J., Keane T.J., Kan S., Rider W.D., Fryer C.J. (1981). Radiation pneumonitis following large single dose irradiation: A re-evaluation based on absolute dose to lung. Int. J. Radiat. Oncol. Biol. Phys..

[2] Fryer C.J., Fitzpatrick P.J., Rider W.D., Poon P. (1978). Radiation pneumonitis: Experience following a large single dose of radiation. Int. J. Radiat. Oncol. Biol. Phys..

[3] Sullivan J.M., Prasanna P.G., Grace M.B., Wathen L.K., Wallace R.L., Koerner J.F., Coleman C.N. (2013). Assessment of biodosimetry methods for a mass-casualty radiological incident: Medical response and management considerations. Health Phys..

[4] Laiakis E.C., Wang Y.W., Young E.F., Harken A.D., Xu Y., Smilenov L., Garty G.Y., Brenner D.J., Fornace A.J. (2017). Metabolic Dysregulation after Neutron Exposures Expected from an Improvised Nuclear Device. Radiat. Res..

[5] Jones J.W., Scott A.J., Tudor G., Xu P.T., Jackson I.L., Vujaskovic Z., Booth C., MacVittie T.J., Ernst R.K., Kane M.A. (2014). Identification and quantitation of biomarkers for radiation-induced injury via mass spectrometry. Health Phys..

[6] Singh V.K., Newman V.L., Romaine P.L., Hauer-Jensen M., Pollard H.B. (2016). Use of biomarkers for assessing radiation injury and efficacy of countermeasures. Expert Rev. Mol. Diagn..

[7] Coy S.L., Cheema A.K., Tyburski J.B., Laiakis E.C., Collins S.P., Fornace A. (2011). Radiation metabolomics and its potential in biodosimetry. Int. J. Radiat. Biol..

[8] Chen Z., Coy S.L., Pannkuk E.L., Laiakis E.C., Fornace A.J., Vouros P. (2018). Differential Mobility Spectrometry-Mass Spectrometry (DMS-MS) in Radiation Biodosimetry: Rapid and High-Throughput Quantitation of Multiple Radiation Biomarkers in Nonhuman Primate Urine. J. Am. Soc. Mass Spectrom..

[9] Pannkuk E.L., Fornace A.J., Laiakis E.C. (2017). Metabolomic applications in radiation biodosimetry: Exploring radiation effects through small molecules. Int. J. Radiat. Biol..

[10] Garty G., Xu Y., Elliston C., Marino S.A., Randers-Pehrson G., Brenner D.J. (2017). Mice and the A-Bomb: Irradiation Systems for Realistic Exposure Scenarios. Radiat. Res..

[11] Xu Y., Randers-Pehrson G., Turner H.C., Marino S.A., Geard C.R., Brenner D.J., Garty G. (2015). Accelerator-Based Biological Irradiation Facility Simulating Neutron Exposure from an Improvised Nuclear Device. Radiat. Res..

[12] Laiakis E.C., Canadell M.P., Grilj V., Harken A.D., Garty G.Y., Astarita G., Brenner D.J., Smilenov L., Fornace A.J. (2019). Serum lipidomic analysis from mixed neutron/X-ray radiation fields reveals a hyperlipidemic and pro-inflammatory phenotype. Sci. Rep..

[13] Johnson C.H., Patterson A.D., Krausz K.W., Lanz C., Kang D.W., Luecke H., Gonzalez F.J., Idle J.R. (2011). Radiation metabolomics. 4. UPLC-ESI-QTOFMS-Based metabolomics for urinary biomarker discovery in gamma-irradiated rats. Radiat. Res..

[14] Tyburski J.B., Patterson A.D., Krausz K.W., Slavik J., Fornace A.J., Gonzalez F.J., Idle J.R. (2009). Radiation metabolomics. 2. Dose- and time-dependent urinary excretion of deaminated purines and pyrimidines after sublethal gamma-radiation exposure in mice. Radiat. Res..

[15] Pilib O.B., Vaitheesvaran B., Saha S., Hartil K., Chen E.I., Goldman D., Fleming W.H., Kurland I.J., Guha C., Golden A. (2015). Intestinal microbiota-derived metabolomic blood plasma markers for prior radiation injury. Int. J. Radiat. Oncol. Biol. Phys..

[16] Manna S.K., Krausz K.W., Bonzo J.A., Idle J.R., Gonzalez F.J. (2013). Metabolomics reveals aging-associated attenuation of noninvasive radiation biomarkers in mice: Potential role of polyamine catabolism and incoherent DNA damage-repair. J. Proteome Res..

[17] Laiakis E.C., Nishita D., Bujold K., Jayatilake M.M., Bakke J., Gahagen J., Authier S., Chang P., Fornace A.J. (2019). Salivary Metabolomics of Total Body Irradiated Nonhuman Primates Reveals Long-Term Normal Tissue Responses to Radiation. Int. J. Radiat. Oncol. Biol. Phys..

[18] Pannkuk E.L., Laiakis E.C., Fornace A.J., Fatanmi O.O., Singh V.K. (2018). A Metabolomic Serum Signature from Nonhuman Primates Treated with a Radiation Countermeasure, Gamma-tocotrienol, and Exposed to Ionizing Radiation. Health Phys..

[19] Tang X., Zheng M., Zhang Y., Fan S., Wang C. (2013). Estimation value of plasma amino acid target analysis to the acute radiation injury early triage in the rat model. Metabolomics.

[20] Pannkuk E.L., Laiakis E.C., Authier S., Wong K., Fornace A.J. (2016). Targeted Metabolomics of Nonhuman Primate Serum after Exposure to Ionizing Radiation: Potential Tools for High-throughput Biodosimetry. RSC Adv..

[21] Moren L., Wibom C., Bergstrom P., Johansson M., Antti H., Bergenheim A.T. (2016). Characterization of the serum metabolome following radiation treatment in patients with high-grade gliomas. Radiat. Oncol..

[22] Pannkuk E.L., Laiakis E.C., Authier S., Wong K., Fornace A.J. (2017). Gas Chromatography/Mass Spectrometry Metabolomics of Urine and Serum from Nonhuman Primates Exposed to Ionizing Radiation: Impacts on the Tricarboxylic Acid Cycle and Protein Metabolism. J. Proteome Res..

[23] Goudarzi M., Weber W.M., Mak T.D., Chung J., Doyle-Eisele M., Melo D.R., Brenner D.J., Guilmette R.A., Fornace A.J. (2015). Metabolomic and lipidomic analysis of serum from mice exposed to an internal emitter, cesium-137, using a shotgun LC-MS(E) approach. J. Proteome Res..

[24] Goudarzi M., Weber W.M., Mak T.D., Chung J., Doyle-Eisele M., Melo D.R., Strawn S.J., Brenner D.J., Guilmette R.A., Fornace A.J. (2015). A Comprehensive Metabolomic Investigation in Urine of Mice Exposed to Strontium-90. Radiat. Res..

[25] Lanz C., Ledermann M., Slavik J., Idle J.R. (2011). The production and composition of rat sebum is unaffected by 3 Gy gamma radiation. Int. J. Radiat. Biol..

[26] Goudarzi M., Weber W.M., Chung J., Doyle-Eisele M., Melo D.R., Mak T.D., Strawn S.J., Brenner D.J., Guilmette R., Fornace A.J. (2015). Serum Dyslipidemia Is Induced by Internal Exposure to Strontium-90 in Mice, Lipidomic Profiling Using a Data-Independent Liquid Chromatography-Mass Spectrometry Approach. J. Proteome Res..

[27] Liu H., Wang Z., Zhang X., Qiao Y., Wu S., Dong F., Chen Y. (2013). Selection of candidate radiation biomarkers in the serum of rats exposed to gamma-rays by GC/TOFMS-based metabolomics. Radiat. Prot. Dosim..

[28] Jones J.W., Jackson I.L., Vujaskovic Z., Kaytor M.D., Kane M.A. (2017). Targeted Metabolomics Identifies Pharmacodynamic Biomarkers for BIO 300 Mitigation of Radiation-Induced Lung Injury. Pharm. Res..

[29] Pannkuk E.L., Laiakis E.C., Mak T.D., Astarita G., Authier S., Wong K., Fornace A.J. (2016). A Lipidomic and Metabolomic Serum Signature from Nonhuman Primates Exposed to Ionizing Radiation. Metabolomics.

[30] Cheema A.K., Suman S., Kaur P., Singh R., Fornace A.J., Datta K. (2014). Long-term differential changes in mouse intestinal metabolomics after gamma and heavy ion radiation exposure. PLoS ONE.

[31] Kurland I.J., Broin P.O., Golden A., Su G., Meng F., Liu L., Mohney R., Kulkarni S., Guha C. (2015). Integrative Metabolic Signatures for Hepatic Radiation Injury. PLoS ONE.

[32] Ros-Mazurczyk M., Wojakowska A., Marczak L., Polanski K., Pietrowska M., Jelonek K., Dominczyk I., Hajduk A., Rutkowski T., Skladowski K. (2017). Ionizing radiation affects profile of serum metabolites: Increased level of 3-hydroxybutyric acid in serum of cancer patients treated with radiotherapy. Acta Biochim. Pol..

[33] Lanz C., Patterson A.D., Slavik J., Krausz K.W., Ledermann M., Gonzalez F.J., Idle J.R. (2009). Radiation metabolomics. 3. Biomarker discovery in the urine of gamma-irradiated rats using a simplified metabolomics protocol of gas chromatography-mass spectrometry combined with random forests machine learning algorithm. Radiat. Res..

[34] Pannkuk E.L., Laiakis E.C., Girgis M., Dowd S.E., Dhungana S., Nishita D., Bujold K., Bakke J., Gahagen J., Authier S. (2019). Temporal Effects on Radiation Responses in Nonhuman Primates: Identification of Biofluid Small Molecule Signatures by Gas Chromatography-Mass Spectrometry Metabolomics. Metabolites.

[35] Jones J.W., Clifford Z., Li F., Tudor G.L., Farese A.M., Booth C., MacVittie T.J., Kane M.A. (2019). Targeted Metabolomics Reveals Metabolomic Signatures Correlating Gastrointestinal Tissue to Plasma in a Mouse Total-body Irradiation Model. Health Phys..

[36] Lee D.Y., Bowen B.P., Nguyen D.H., Parsa S., Huang Y., Mao J.H., Northen T.R. (2012). Low-dose ionizing radiation-induced blood plasma metabolic response in a diverse genetic mouse population. Radiat. Res..

[37] Ghosh S.P., Singh R., Chakraborty K., Kulkarni S., Uppal A., Luo Y., Kaur P., Pathak R., Kumar K.S., Hauer-Jensen M. (2013). Metabolomic changes in gastrointestinal tissues after whole body radiation in a murine model. Mol. Biosyst..

[38] Khan A.R., Rana P., Devi M.M., Chaturvedi S., Javed S., Tripathi R.P., Khushu S. (2011). Nuclear magnetic resonance spectroscopy-based metabonomic investigation of biochemical effects in serum of gamma-irradiated mice. Int. J. Radiat. Biol..

[39] Wibom C., Surowiec I., Moren L., Bergstrom P., Johansson M., Antti H., Bergenheim A.T. (2010). Metabolomic patterns in glioblastoma and changes during radiotherapy: A clinical microdialysis study. J. Proteome Res..

[40] Goudarzi M., Mak T.D., Chen C., Smilenov L.B., Brenner D.J., Fornace A.J. (2014). The effect of low dose rate on metabolomic response to radiation in mice. Radiat. Environ. Biophys..

[41] Goudarzi M., Weber W., Mak T.D., Chung J., Doyle-Eisele M., Melo D., Brenner D.J., Guilmette R.A., Fornace A.J. (2014). Development of urinary biomarkers for internal exposure by cesium-137 using a metabolomics approach in mice. Radiat. Res..

[42] Chen C., Brenner D.J., Brown T.R. (2011). Identification of urinary biomarkers from X-irradiated mice using NMR spectroscopy. Radiat. Res..

[43] Tyburski J.B., Patterson A.D., Krausz K.W., Slavik J., Fornace A.J., Gonzalez F.J., Idle J.R. (2008). Radiation metabolomics. 1. Identification of minimally invasive urine biomarkers for gamma-radiation exposure in mice. Radiat. Res..

[44] Goudarzi M., Mak T.D., Jacobs J.P., Moon B.H., Strawn S.J., Braun J., Brenner D.J., Fornace A.J., Li H.H. (2016). An Integrated Multi-Omic Approach to Assess Radiation Injury on the Host-Microbiome Axis. Radiat. Res..

[45] Mak T.D., Tyburski J.B., Krausz K.W., Kalinich J.F., Gonzalez F.J., Fornace A.J. (2015). Exposure to ionizing radiation reveals global dose- and time-dependent changes in the urinary metabolome of rat. Metabolomics.

[46] Johnson C.H., Patterson A.D., Krausz K.W., Kalinich J.F., Tyburski J.B., Kang D.W., Luecke H., Gonzalez F.J., Blakely W.F., Idle J.R. (2012). Radiation metabolomics. 5. Identification of urinary biomarkers of ionizing radiation exposure in nonhuman primates by mass spectrometry-based metabolomics. Radiat. Res..

[47] Pannkuk E.L., Laiakis E.C., Authier S., Wong K., Fornace A.J. (2015). Global Metabolomic Identification of Long-Term Dose-Dependent Urinary Biomarkers in Nonhuman Primates Exposed to Ionizing Radiation. Radiat. Res..

[48] Pannkuk E.L., Laiakis E.C., Gill K., Jain S.K., Mehta K.Y., Nishita D., Bujold K., Bakke J., Gahagen J., Authier S. (2019). Liquid Chromatography-Mass Spectrometry-Based Metabolomics of Nonhuman Primates after 4 Gy Total Body Radiation Exposure: Global Effects and Targeted Panels. J. Proteome Res..

[49] Jones J.W., Tudor G., Li F., Tong Y., Katz B., Farese A.M., MacVittie T.J., Booth C., Kane M.A. (2015). Citrulline as a Biomarker in the Murine Total-Body Irradiation Model: Correlation of Circulating and Tissue Citrulline to Small Intestine Epithelial Histopathology. Health Phys..

[50] Jones J.W., Tudor G., Bennett A., Farese A.M., Moroni M., Booth C., MacVittie T.J., Kane M.A. (2014). Development and validation of a LC-MS/MS assay for quantitation of plasma citrulline for application to animal models of the acute radiation syndrome across multiple species. Anal. Bioanal. Chem..

[51] Goudarzi M., Chauthe S., Strawn S.J., Weber W.M., Brenner D.J., Fornace A.J. (2016). Quantitative Metabolomic Analysis of Urinary Citrulline and Calcitroic Acid in Mice after Exposure to Various Types of Ionizing Radiation. Int. J. Mol. Sci..

[52] Jones J.W., Alloush J., Sellamuthu R., Chua H.L., MacVittie T.J., Orschell C.M., Kane M.A. (2019). Effect of Sex on Biomarker Response in a Mouse Model of the Hematopoietic Acute Radiation Syndrome. Health Phys..

[53] Shim S., Jang W.S., Lee S.J., Jin S., Kim J., Lee S.S., Bang H.Y., Jeon B.S., Park S. (2014). Development of a new minipig model to study radiation-induced gastrointestinal syndrome and its application in clinical research. Radiat. Res..

[54] Jones J.W., Bennett A., Carter C.L., Tudor G., Hankey K.G., Farese A.M., Booth C., MacVittie T.J., Kane M.A. (2015). Citrulline as a Biomarker in the Non-human Primate Total- and Partial-body Irradiation Models: Correlation of Circulating Citrulline to Acute and Prolonged Gastrointestinal Injury. Health Phys..

[55] Onal C., Kotek A., Unal B., Arslan G., Yavuz A., Topkan E., Yavuz M. (2011). Plasma citrulline levels predict intestinal toxicity in patients treated with pelvic radiotherapy. Acta Oncol..

[56] Laiakis E.C., Hyduke D.R., Fornace A.J. (2012). Comparison of mouse urinary metabolic profiles after exposure to the inflammatory stressors gamma radiation and lipopolysaccharide. Radiat. Res..

[57] Laiakis E.C., Strassburg K., Bogumil R., Lai S., Vreeken R.J., Hankemeier T., Langridge J., Plumb R.S., Fornace A.J., Astarita G. (2014). Metabolic phenotyping reveals a lipid mediator response to ionizing radiation. J. Proteome Res..

[58] Laiakis E.C., Mak T.D., Anizan S., Amundson S.A., Barker C.A., Wolden S.L., Brenner D.J., Fornace A.J. (2014). Development of a metabolomic radiation signature in urine from patients undergoing total body irradiation. Radiat. Res..

[59] Roszkowski K., Gackowski D., Rozalski R., Dziaman T., Siomek A., Guz J., Szpila A., Foksinski M., Olinski R. (2008). Small field radiotherapy of head and neck cancer patients is responsible for oxidatively damaged DNA/oxidative stress on the level of a whole organism. Int. J. Cancer.

[60] Jelonek K., Pietrowska M., Ros M., Zagdanski A., Suchwalko A., Polanska J., Marczyk M., Rutkowski T., Skladowski K., Clench M.R. (2014). Radiation-induced changes in serum lipidome of head and neck cancer patients. Int. J. Mol. Sci..

[61] Lin X., Fuks Z., Kolesnick R. (2000). Ceramide mediates radiation-induced death of endothelium. Crit. Care Med..

[62] Kolesnick R., Fuks Z. (2003). Radiation and ceramide-induced apoptosis. Oncogene.

[63] Jones J.W., Carter C.L., Li F., Yu J., Pierzchalski K., Jackson I.L., Vujaskovic Z., Kane M.A. (2017). Ultraperformance convergence chromatography-high resolution tandem mass spectrometry for lipid biomarker profiling and identification. Biomed. Chromatogr..

[64] Carter C.L., Jones J.W., Farese A.M., MacVittie T.J., Kane M.A. (2017). Lipidomic dysregulation within the lung parenchyma following whole-thorax lung irradiation: Markers of injury, inflammation and fibrosis detected by MALDI-MSI. Sci. Rep..

[65] Laiakis E.C., Pannkuk E.L., Chauthe S.K., Wang Y.W., Lian M., Mak T.D., Barker C.A., Astarita G., Fornace A.J. (2017). A Serum Small Molecule Biosignature of Radiation Exposure from Total Body Irradiated Patients. J. Proteome Res..

[66] Pannkuk E.L., Laiakis E.C., Singh V.K., Fornace A.J. (2017). Lipidomic Signatures of Nonhuman Primates with Radiation-Induced Hematopoietic Syndrome. Sci. Rep..

[67] Williams J.P., McBride W.H. (2011). After the bomb drops: A new look at radiation-induced multiple organ dysfunction syndrome (MODS). Int. J. Radiat. Biol..

[68] MacVittie T.J., Farese A.M., Kane M.A. (2019). ARS, DEARE, and Multiple-organ Injury: A Strategic and Tactical Approach to Link Radiation Effects, Animal Models, Medical Countermeasures, and Biomarker Development to Predict Clinical Outcome. Health Phys..

[69] Hall E.J., Giaccia A.J. (2019). Radiobiology for the Radiologist.

[70] Robbins M.E., Zhao W. (2004). Chronic oxidative stress and radiation-induced late normal tissue injury: A review. Int. J. Radiat. Biol..

[71] Zhao W., Robbins M.E. (2009). Inflammation and chronic oxidative stress in radiation-induced late normal tissue injury: Therapeutic implications. Curr. Med. Chem..

[72] Eccles L.J., O’Neill P., Lomax M.E. (2011). Delayed repair of radiation induced clustered DNA damage: Friend or foe?. Mutat. Res. Mol. Mech. Mutagen..

[73] Benderitter M., Vincent-Genod L., Pouget J.P., Voisin P. (2003). The cell membrane as a biosensor of oxidative stress induced by radiation exposure: A multiparameter investigation. Radiat. Res..

[74] Davies M.J. (2005). The oxidative environment and protein damage. Biochim. Biophys. Acta.

[75] Clemens M.R., Ladner C., Schmidt H., Ehninger G., Einsele H., Bühler E., Waller H.D., Gey K.F. (1989). Decreased essential antioxidants and increased lipid hydroperoxides following high-dose radiochemotherapy. Free Radic. Res. Commun..

[76] Jelonek K., Pietrowska M., Widlak P. (2017). Systemic effects of ionizing radiation at the proteome and metabolome levels in the blood of cancer patients treated with radiotherapy: The influence of inflammation and radiation toxicity. Int. J. Radiat. Biol..

[77] Gross N.J. (1977). Pulmonary effects of radiation therapy. Ann. Intern. Med..

[78] Carter C.L., Jones J.W., Barrow K., Kieta K., Taylor-Howell C., Kearney S., Smith C.P., Gibbs A., Farese A.M., MacVittie T.J. (2015). A MALDI-MSI Approach to the Characterization of Radiation-Induced Lung Injury and Medical Countermeasure Development. Health Phys..

[79] Rustam Y.H., Reid G.E. (2018). Analytical Challenges and Recent Advances in Mass Spectrometry Based Lipidomics. Anal. Chem..

[80] Gatto L., Hansen K.D., Hoopmann M.R., Hermjakob H., Kohlbacher O., Beyer A. (2016). Testing and Validation of Computational Methods for Mass Spectrometry. J. Proteome Res..

[81] Mittendorfer B. (2005). Sexual Dimorphism in Human Lipid Metabolism. J. Nutr..

[82] Williamson J.R., Cooper R.H. (1980). Regulation of the citric acid cycle in mammalian systems. FEBS Lett..

[83] Azzam E.I., Jay-Gerin J.P., Pain D. (2012). Ionizing radiation-induced metabolic oxidative stress and prolonged cell injury. Cancer Lett..

[84] Pantopoulos K., Hentze M.W. (1995). Rapid responses to oxidative stress mediated by iron regulatory protein. EMBO J..

[85] Tominaga H., Kodama S., Matsuda N., Suzuki K., Watanabe M. (2004). Involvement of reactive oxygen species (ROS) in the induction of genetic instability by radiation. J. Radiat. Res..

[86] Sumien N., Shetty R.A., Gonzales E.B. (2018). Creatine, Creatine Kinase, and Aging. Biochemistry and Cell Biology of Ageing: Part I Biomedical Science.

[87] Haberland G.L., Schreier K., Bruns F., Altman K.I., Hempelmann L.H. (1955). Creatine-creatinine metabolism in radiation myopathy. Nature.

[88] Tarnopolsky M. (2011). Creatine as a therapeutic strategy for myopathies. Amino Acids.

[89] Ghosh P.S., Milone M. (2015). Clinical and laboratory findings of 21 patients with radiation-induced myopathy. J. Neurol. Neurosurg. Psychiatry.

[90] Tarnopolsky M.A., Parshad A., Walzel B., Schlattner U., Wallimann T. (2001). Creatine transporter and mitochondrial creatine kinase protein content in myopathies. Muscle Nerve.

[91] Guipaud O., Benderitter M. (2009). Protein biomarkers for radiation exposure: Towards a proteomic approach as a new investigation tool. Ann. Ist. Super. sanitÃ.

[92] Kaur A., Ten Have G.A.M., Hritzo B., Deutz N.E.P., Olsen C., Moroni M. (2020). Morphological and functional impairment in the gut in a partial body irradiation minipig model of GI-ARS. Int. J. Radiat. Biol..

[93] Lutgens L., Lambin P. (2007). Biomarkers for radiation-induced small bowel epithelial damage: An emerging role for plasma Citrulline. World J. Gastroenterol..

[94] Wakabayashi Y., Yamada E., Yoshida T., Takahashi N. (1995). Effect of intestinal resection and arginine-free diet on rat physiology. Am. J. Physiol. Gastrointest. Liver Physiol..

[95] Yang S., He X., Liu Y., Ding X., Jiang H., Tan Y., Lu H. (2019). Prognostic Significance of Serum Uric Acid and Gamma-Glutamyltransferase in Patients with Advanced Gastric Cancer. Dis. Markers.

[96] Scheepers L.E., Wei F., Stolarz-Skrzypek K., Malyutina S., Tikhonoff V., Thijs L., Salvi E., Barlassina C., Filipovský J., Casiglia E. (2016). THU0519 Xanthine Oxidase Gene Variants and Their Association with Blood Pressure and Incident Hypertension: A Population Study. Ann. Rheum. Dis..

[97] Paithankar J.G., Kudva A.K., Raghu S.V., Patil R.K. (2020). Radioprotective role of uric acid: Evidence from studies in Drosophila and human dermal fibroblast cells. Mol. Biol. Rep..

[98] Quehenberger O., Armando A.M., Brown A.H., Milne S.B., Myers D.S., Merrill A.H., Bandyopadhyay S., Jones K.N., Kelly S., Shaner R.L. (2010). Lipidomics reveals a remarkable diversity of lipids in human plasma. J. Lipid Res..

[99] Schiller J., Fuchs B., Arnhold J., Arnold K. (2003). Contribution of reactive oxygen species to cartilage degradation in rheumatic diseases: Molecular pathways, diagnosis and potential therapeutic strategies. Curr. Med. Chem..

[100] Wolrab D., Jirásko R., Chocholoušková M., Peterka O., Holčapek M. (2019). Oncolipidomics: Mass spectrometric quantitation of lipids in cancer research. TrAC Trends Anal. Chem..

[101] Haimovitz-Friedman A., Kan C.C., Ehleiter D., Persaud R.S., McLoughlin M., Fuks Z., Kolesnick R.N. (1994). Ionizing radiation acts on cellular membranes to generate ceramide and initiate apoptosis. J. Exp. Med..

[102] Rotolo J., Stancevic B., Zhang J., Hua G., Fuller J., Yin X., Haimovitz-Friedman A., Kim K., Qian M., Cardo-Vila M. (2012). Anti-ceramide antibody prevents the radiation gastrointestinal syndrome in mice. J. Clin. Investig..

[103] Hao D., Sarfaraz M.O., Farshidfar F., Bebb D.G., Lee C.Y., Card C.M., David M., Weljie A.M. (2016). Temporal characterization of serum metabolite signatures in lung cancer patients undergoing treatment. Metabolomics.

[104] Mak T.D., Laiakis E.C., Goudarzi M., Fornace A.J. (2015). Selective paired ion contrast analysis: A novel algorithm for analyzing postprocessed LC-MS metabolomics data possessing high experimental noise. Anal. Chem..

[105] Gagnebin Y., Tonoli D., Lescuyer P., Ponte B., de Seigneux S., Martin P.Y., Schappler J., Boccard J., Rudaz S. (2017). Metabolomic analysis of urine samples by UHPLC-QTOF-MS: Impact of normalization strategies. Anal. Chim. Acta.

